# Wrapping up eight years of editing in a healthy medical journal

**DOI:** 10.1111/aogs.14511

**Published:** 2023-02-24

**Authors:** Nils‐Halvdan Morken

**Affiliations:** ^1^ Department of Clinical Science University of Bergen Bergen Norway; ^2^ Department of Obstetrics and Gynecology Haukeland University Hospital Bergen Norway

AOGS is an old scientific journal in obstetrics and gynecology and has a history that spans back more than 100 years. In fact, we planned for a large celebration of its 100 years anniversary in 2021 at the Nordic Congress in Obstetrics and Gynecology in Reykjavik, but as with many other planned celebrations during 2020 and 2021, it ended in a digital event. At least, we managed to mark AOGS 100 years of serving the scientific community, however without the real‐life dimension that is so important for all living creatures.

Quant geeks, like us, love all that is quantifiable and medical journals tend to be evaluated and appreciated by their journal impact factor. This goes for the whole scientific community, submitting authors at all stages, editors, funding agencies, universities, and public authorities. A lot has been written about this metric and it is discussed whether it measures true impact, impact on life and people and impact on treatment of our patients. As recently stated by a fellow scientist: “The journal impact factor is wrong in so many ways, but it is so easy.”^1^

A few years ago, a correspondence between editors of *Epidemiology* and *The International Journal of Epidemiology* was sparked by an editorial in *Epidemiology*, where it was suggested that the impact factor did not influence where authors choose to submit papers.^2^ The conclusion by the editorial team of *Epidemiology* was based on a before‐and‐after comparison of the ratio of submissions in the second half of 2011 to those in the first half, as impact factors are released typically in June each year. The editors of *International Journal of Epidemiology* responded by showing a close relation between their impact factor as released in June of each year by number of submissions to the journal in the subsequent year.^3^ This led the editors in *Epidemiology* to do the same calculations on their numbers, finding a similar pattern, with rising impact factor and number of submissions. However, the editors of *Epidemiology* were not quite sure what to make of this ecological association in their reply. It could be a causal effect of impact factor on submissions, as interpreted by the editors of *International Journal of Epidemiology*, or that the two trends may simply reflect the natural growth of healthy journals.^3^

If we do the same with the AOGS numbers, plotting impact factor by year and number of submissions (summarized from July to June), we see a somewhat similar pattern. The impact factor has had a steady increase and we must admit that also the editorial board of AOGS has been pleased by the increase over the last years, rising from 2.426 in 2014 to 4.544 in 2021. Currently, AOGS is ranked number 15 among 85 journals within obstetrics and gynecology. At the same time, submissions have shown an increasing tendency, with a clear corona‐bump in 2020. Another notable figure is the slightly reduced percentage of acceptance over these years. We probably had more papers to select the best publishable from. To those who think that AOGS is a regional journal, I must disappoint you. Submissions come from all over the world, so keep submitting to our international journal. For the relevant numbers, please see Table 1.

**TABLE 1:** Annual submissions, percent accepted, origin of submissions by region, Impact Factor, and submissions from July to June following the annual Impact Factor, AOGS, 2014 to 2021

	2014	2015	2016	2017	2018	2019	2020	2021
Annual submissions	1030	1022	934	927	1034	1217	1603	1270
Percent accepted	23%	21%	22%	21%	23%	20%	16%	15%
Origin of submissions								
Nordic^a^	21%	23%	26%	22%	24%	17%	13%	16%
Other European	24%	26%	26%	24%	24%	27%	24%	22%
Asia	37%	33%	31%	30%	35%	36%	42%	48%
English speaking^b^	11%	11%	10%	14%	12%	12%	12%	9%
Latin America	3%	4%	4%	5%	4%	3%	5%	2%
Africa	4%	3%	3%	5%	1%	5%	4%	3%
Impact factor	2.426	2.191	2.480	2.649	2.741	2.770	3.636	4.544
Submissions July to June	2014–15	2015–16	2016–17	2017–18	2018–19	2019–20	2020–21	
	1063	987	888	953	1156	1411	1567	n. a.

^a^ Sweden, Denmark, Finland, Norway and Iceland.

^b^ USA, UK, Canada, Australia and New Zealand.

I have over the last 8 years had the pleasure of serving at the editorial board of AOGS, as an associate editor appointed by the Norwegian Society of Gynecology and Obstetrics. The journal is owned by the Nordic Federation of Associations in Obstetrics and Gynecology (NFOG) and each of the five member countries appoint editors to AOGS for a maximum period of 8 years. My watch has now come to an end, and it is time to wrap up and reflect. First, in my opinion, the limited time‐period for associate editors is one important factor in a healthy medical journal. By this rule, the NFOG has assured that people do not stay forever, and that new ideas and opinions are allowed to flow continuously into the journal's editorial board.

It has for me personally been a joyful journey with a steep learning curve that would have been impossible without all the people I have collaborated with and met over the past 8 years. People and their relations are in my opinion also a crucial factor in the development of a healthy medical journal. First and foremost, thanks to our chief editor Ganesh Acharya for being such an inspiring and involving boss. You are dedicated with an ability to see people and combine scientific toughness with a kind and mild personality that truly believes in people. Advice is given without offending, combined with an ability to seek advice when needed. You have managed to create a friendly environment on the board and arranged inspiring learning sessions, involving external capacities that both have enlightened us and given food for thought. All this has inspired me and others to work harder.

Thanks to all fellow associate editors and international collaborators over the years. We have always managed to have a good tone at meetings, creative learning sessions, inspiring and fruitful discussions, and not to forget our social gatherings with laughter and good meals together. Then of course, thanks to all colleagues around the globe I have asked to review manuscripts, sometimes at inconvenient times and thanks to all authors that have sent quality papers to AOGS. Without you there would be no AOGS! Clearly, even a scientific journal, based on reporting of rigorous facts and truth is about people, their relations, creative collaboration, and about being critical friends. The latter may though be controversial for some, at a time when impartiality is the word of the day. Value of relations, collaboration and extensive and rigorous review efforts are difficult to measure and count. Reductionists like us are reluctant when things in life cannot be measured and given a number that preferably should be significant, with a *p*‐value <0.05. To me it does not matter who said: “Not everything that can be counted counts and not everything that counts can be counted,” because it bears a truth whenever life and people are involved, also for a healthy scientific journal, like AOGS.
